# The association between generalized joint hypermobility and active horizontal shoulder abduction in 10–15 year old competitive swimmers

**DOI:** 10.1186/s13102-016-0044-y

**Published:** 2016-07-12

**Authors:** Tina Junge, Peter Henriksen, Heidi Lund Andersen, Linette Dyg Byskov, Hans Kromann Knudsen, Birgit Juul-Kristensen

**Affiliations:** Department of Physiotherapy, University College Lillebaelt, Odense, Denmark; Health Sciences Research Centre, University College Lillebaelt, Odense, Denmark; Department of Sports Science and Clinical Biomechanics, University of Southern Denmark, Odense, Denmark; Institute of Occupational Therapy, Physiotherapy and Radiography, Bergen University College, Bergen, Norway

**Keywords:** Competitive swimmers, Generalized joint hypermobility, Active horizontal shoulder abduction, Shoulder injuries

## Abstract

**Background:**

Increased shoulder mobility and Generalised Joint Hypermobility (GJH) are assumed to be predisposing risk factors for shoulder injuries. The association between GJH and shoulder mobility among competitive swimmers is unknown. The aim was to study the association between GJH and active horizontal shoulder abduction (AHSA) in young, competitive swimmers and to describe normative values of AHSA in this group.

**Methods:**

In total, 92 swimmers (10–15 years) without shoulder pain participated. GJH was evaluated with the Beighton Tests (BT) for joint hypermobility. Shoulder mobility was measured as maximum AHSA. A multiple regression model was used to assess associations between GJH and AHSA.

**Results:**

Overall, positive associations were found between GJH and AHSA. An increase of BT score was associated with an increase of AHSA, seen as an increased AHSA of 3.9°, 5.7° and 7.9° by BT cut off points ≥5/9, ≥6/9 and ≥7/9, respectively. Normative values for AHSA ranged from 40° to 52°, depending on age.

**Conclusions:**

Positive associations were found between GJH and AHSA, as maximum AHSA range increased with increasing BT scores. Due to lack of shoulder mobility tests in the BT scoring system, the AHSA test seems to be a promising supplemental test.

## Background

A competitive swimmer swims 9–110 km a week, depending on age, fitness level and training requirements [1]. This amount leads to a large amount of repetitive movements, which may result in overuse injuries of the shoulder [2]. Although competitive swimming attracts a large number of children and adolescents, there are almost no epidemiological studies on adolescent shoulder injuries for children or adolescents within this sport [3]. The prevalence of shoulder injuries in adult competitive swimmers varies from 40 to 91 %, partly due to inconsistency in definition of injury, study design and data collection methods [2].

Freestyle stroke is a whole body movement, but with the upper extremities producing 90 % of the propulsive power [4], requiring shoulder strength, endurance and stability. Biomechanical challenges of the shoulder for the freestyle stroke may occur during the recovery phase, which demands a large Range Of Motion (ROM). A large ROM is anticipated to be advantageous by allowing the swimmer to achieve a body position that permits a larger stroke length, positively associated with swimming speed [2]. Consequently, the shoulder is repetitively forced into extension, horizontal abduction and internal rotation. Thus, swimming induces a large amount of stress on the anterior capsule and ligaments of the glenohumeral joint, which may lead to local shoulder laxity and/or an increased shoulder ROM [2, 5].

Increased shoulder ROM may be a genetic predisposition or an adaptation to the repetitive and excessive overhead movements in swimming. A condition in individuals with hereditary increased ROM is Generalised Joint Hypermobility (GJH), in which the ligaments and capsules are more lax compared with the normal population [6]. GJH is frequently present in swimmers, since as many as 20 % of male and female adult swimmers are classified with GJH [7]. Also, a higher prevalence of GJH is found in young, competitive swimmers compared with non-competitive swimmers [5, 8]. Increased shoulder ROM alone and GJH including shoulder hypermobility may play an important role in development of shoulder pain and injuries, and the extent of both conditions must be described as part of an injury sequence prevention strategy, focusing on underlying mechanisms [9].

GJH is most often assessed by the Beighton Tests (BT), involving a score from 0 to 9, with a suggested cut off point for classification of GJH of ≥4/9 in adults [10]. A cut off point for classifying children or adolescents with GJH has not been established, which is why the cut off point for GJH varies between studies [8, 11, 12]. The BT for hypermobility includes the 1st and 5th fingers, the elbows, the knees and forward bending, but there is no test for shoulder hypermobility [6]. The Active Horizontal Shoulder Abduction test (AHSA) (Illustration 1) could be a relevant shoulder mobility test for swimmers, simulating a swim-related stressful situation like the recovery phase of the freestyle stroke. Since the BT score is closely related to the general mobility of all joints [6] [13], the AHSA as supplemental test may provide a more comprehensive profile of the single swimmer’s shoulder joint mobility.

In order to compare AHSA between different populations, normative data is required for young, competitive swimmers, taking into account age and sex. Such data exists for 15–21 year olds [14], however, no reference material covering younger swimmers between the ages of 10 to 15 years.

Thus, the aims of this study were to examine the association between shoulder ROM, defined by the Horizontal Shoulder Abduction test (AHSA) and the BT score, and to present normative values for AHSA in young, competitive swimmers between the ages of 10–15 years.

## Methods

### Participants

In total, 116 young, competitive swimmers aged 10–15 years were invited to take part in the current study, a substudy of the longitudinal cohort study, the *“Physical Performance in Young Competitive Swimmers”* (PPYCS). PPYCS involves young, competitive swimmers from 5 different swim teams at Funen, Denmark. The overall purpose of PPYCS is to assess development of mobility, physical fitness, motor performance and sports-related injuries longitudinally, within this specific cohort.

The inclusion criteria were healthy swimmers aged 10–15 years, participating in competitive swim teams, defined as a swimmer taking part in competitions at a level corresponding to a local district, a larger region or at national level. Exclusion criteria were musculoskeletal pain, injury or illness preventing the swimmer from participating on the day of testing.

### Testers

Testers were last semester physiotherapy students, who carried out this study as part of their final thesis. Prior to testing, the testers undertook a supervised training phase by two experienced physiotherapists (HKK & TJ) in order to standardise test instructions and procedures and to approve a satisfactory inter- and intra tester reproducibility. The training phase included testing of approximately 100 adolescents aged 10–18 years. For both the BT and the AHSA test, the testers gave instructions to the swimmers according to the standardized protocol, along with visual demonstrations. The testers performing the AHSA test were blinded to the status of the swimmers being GJH or controls. All swimmers were tested with the BT and afterwards with the AHSA test.

### Beighton tests (BT)

GJH was evaluated according to the BT score [6, 15]. The BT consists of five manoeuvres: 1) passive dorsiflexion of the little fingers beyond 90°, 2) passive apposition of the thumbs to the flexor aspects of the forearm, 3) hyperextension of the elbows beyond 10°, 4) hyperextension of the knees beyond 10° and 5) forward flexion of the trunk with the knees straight, resting the palms easily on the floor. One point was allocated for each of the tests being positive as described, bilaterally for manoeuvres 1–4, with a total score ranging from 0 to 9 [6]. The reliability of the Beighton score has been examined for the current test procedures in a corresponding age population with good to excellent inter and intra tester reproducibility [15]. Children were classified with GJH at different BT cut off points of ≥5/9, ≥6/9 and ≥7/9. The control group included children with a BT score ≤4/9.

### Test for active horizontal shoulder abduction (AHSA)

AHSA was measured in prone using a Saunder Digital Inclinometer (Illustration 1). Each swimmer was lying in a prone starting position with the upper arm placed horizontally, 90° elbow flexion, fingertips pointing towards the floor and the head turned in the opposite direction of the shoulder being tested to avoid rotation of the spine. The Digital Inclinometer was mounted at the proximal end of an angle hinge, perpendicular to the distal end. The distal end was aligned to the forearm and the proximal to the upper arm, so that the elbow joint was kept in 90° angle, to secure stabilization during measurements and to optimize standardization during measurements (Illustration 2). The swimmer was asked to lift the elbow as far as possible towards the ceiling. The test was performed three times actively for both shoulders, and the maximum angle of the upper arm in horizontal shoulder abduction was recorded in degrees.

**Illustration 1:**
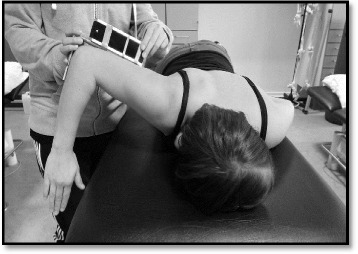
Test of the horizontal shoulder abduction

**Illustration 2:**
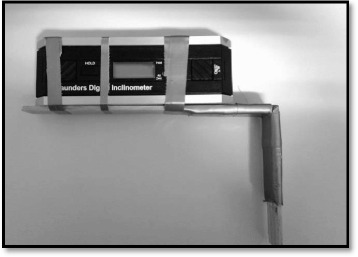
Device with inclinometer

### Data analysis

Data was tested for Normality with Shapiro-Wilks tests. Since no significant difference was found between left and right shoulder in the AHSA test (*p* = 0.09), a mean of AHSA for both shoulders was used for further analyses. A multiple linear regression model was used to test the association between AHSA and the BT score, using the three different BT cut off points at ≥5/9, ≥6/9 and ≥7/9, adjusted for sex and age.

When calculating normative data of AHSA, age was found to be significantly or close to significant associated with the maximum AHSA for cut off point ≥6/9 (*p* = 0.04) and ≥7/9 (*p* = 0.052), for which reason data were stratified by age. There was no statistically significant effect of sex on maximum AHSA, hence data for boys and girls were merged and presented for the entire sample.

A *p*-value of 0.05 or less was considered statistically significant. All statistical analyses were conducted in SPSS version 20 (IBM SPSS Inc, Chicago, IL, USA, 2012).

## Results

In total, 92 young, competitive swimmers (mean age 13 years; 49 girls), with no shoulder pain participated.

In demographic variables, the only significant difference were height, with the GJH group being shorter (Table 1). A larger prevalence of girls was found in the GJH group, no matter the cut-off point (Tables 1 and 2).

**Table 1:** Characteristics of participants with Generalised Joint Hypermobility (GJH), and without GJH (controls)

	GJH (*n* = 29)	Controls (*n* = 63)	*p-value*
	(mean, SD)	(mean, SD)	
Sex (no. girls)	20	29	0.04^a^
Age (years)	12.62 ± 1.6	12.88 ± 1.2	0.37
Height (m)	1.61 ± 11.7	1.66 ± 10.6	0.05^b^
Mass (kg)	50.01 ± 11.7	53.51 ± 10.2	0.14
Right AHSA (degree)	45.55 ± 9.55	43.23 ± 13.6	0.41
Left AHSA (degree)	44.06 ± 9.68	41.03 ± 9.0	0.14

*AHSA* Active Horizontal Shoulder Abduction

^a^Significant difference between sexes

^b^Significant difference between groups

**Table 2:** Prevalence stratified by sex for participants with Generalised Joint Hypermobility (GJH) at cut-off points 5/9, 6/9 and 7/9, and for controls

	Participants (*n*)	GJH (%)	Controls (%)
Cut off point ≥5/9			
Boys	43	9 (21)	34 (37)
Girls	49	20 (41)	29 (31)
Total	92	29 (32)	63 (68)
Cut off point ≥6/9			
Boys	43	5 (12)	38 (41)
Girls	49	14 (29)	35 (38)
Total	92	19 (21)	73 (79)
Cut off point ≥7/9			
Boys	43	3 (7)	40 (43.5)
Girls	49	9 (18)	40 (43.5)
Total	92	12 (13)	80 (87)

Significant positive associations were found between the BT score and AHSA, adjusted for sex and age (Table 3). An increasing degree of AHSA was found with an increasing BT score for all cut-off points. Swimmers with BT at cut-off point ≥5/9 had an AHSA which was 3.98° higher than controls. For the BT score of ≥7/9, the difference in AHSA increased to 7.90° between groups. There was no effect of gender. A significant negative effect on maximum AHSA with age was only found at cut-off point ≥6/9.

**Table 3:** Association of Active Horizontal Shoulder Abduction in degrees and the Beighton tests score by cut off point ≥5/9, ≥6/9 and ≥7/9

Beighton tests score	Estimate (degrees)	95 % CI	*p*-value
Cut off point ≥5/9	3.98	[−0.12–8.07]	0.057
Age	−1.28	[−2.66–0.10]	0.069
Girls	−2.22	[−6.02–1.59]	0.251
Cut off point ≥6/9	5.71	[1.02–10.42]	0.018^a^
Age	−1.43	[−2.80–0.07]	0.040^a^
Girls	−2.49	[−6.27–1.29]	0.194
Cut off point ≥7/9	7.90	[2.27–13.52]	0.007^a^
Age	−1.34	[−2.69–0.01]	0.052
Girls	−2.44	[−6.15–1.26]	0.194

^a^Significant results, with *p*≤0.05

Normative data for the AHSA test were from 40.02° to 51.83° (mean 43.95°) across all age groups, for both controls and at all cut-off points for the GJH group, with the highest value and range of shoulder mobility for the youngest age groups, decreasing by age (Table 4).

**Table 4:** Normative data for the Active Horizontal Shoulder Abduction (AHSA) test in degrees for both the GJH group at all cut off points and controls

Age	Mean ± SD (degree)	95 % CI (degree)
10	51.83 ± 10.05	26.86–76.81
11	45.60 ± 7.19	41.62–49.58
12	40.71 ± 10.22	36.06–45.37
13	43.31 ± 7.73	39.79–46.83
14	40.02 ± 8.11	36.43–43.62
15	42.25 ± 11.32	34.15–50.35

## Discussion

In the current study, a significant positive association was found between GJH and AHSA, with AHSA ranging from 5° to 8° higher than controls, with an increased BT score. Normative data for AHSA ranged from 40° to 52°, depending on age.

Swimmers with GJH ≥7/9 presented with 15 and 20 % higher maximum AHSA mobility than controls at the age of 10 and 14, respectively. These findings are in contrast to a previous study, where no association was found between GJH and shoulder rotation and extension in young swimmers, with only 4 swimmers classified as GJH at cut off point ≥5/9 [16]. Likewise, in non sport-specific adults, there was no association between number of positive BT and passive glenohumeral ROM values [17, 18]. The current data, however, indicates that maximum AHSA is associated with GJH in adolescent swimmers, and that this test may reveal increased shoulder mobility in this group.

Increased ROM of the shoulder is often seen in swimmers, and is generally considered a potential risk factor for shoulder pain and/or injuries. However, such relationship has not yet been confirmed [3, 19–21]. In a cross-sectional study of 32 competitive swimmers aged 15 to 21 years, no association was seen between horizontal abduction and actual shoulder pain, however, classification of GJH was not included in the test battery [14]. Similarly, no association was found between shoulder internal and external ROM and the occurrence of pain in a cross-sectional study including15 participants, with six of these categorised as having shoulder laxity, but none with GJH [22]. The decreased passive stability in GJH along with a higher degree of AHSA requires a large contribution of active stability provided by the rotator cuff muscles to control for glenohumeral translation. Increased demands for active stability may hypothetically result in muscle fatigue, leading to repetitive micro trauma seen as overuse injuries.

At cut off point ≥7/9, maximum AHSA was increased by 8° compared to controls, and a question to be considered is whether GJH and increased shoulder ROM is predictive of shoulder injury and/or pain. The ability to establish a relationship between the BT score including maximum AHSA and shoulder injury in young competitive swimmers is limited by the cross-sectional design of the current study.

The current normative values are in line with a previous study of AHSA in 15–21 year old competitive swimmers [14], however, not tested with BT, but with AHSA presented for the whole group (left shoulder 44° ±14° and right shoulder 44° ±16° versus the current range of 40–51°). The current study included both swimmers with and without GJH in the normative values, representing a typical population of swimmers.

The prevalence of girls at 12 years (mean age) with GJH in the current population of swimmers was about twice as high as found in a normal population (different sports) at similar age; 41 % vs. 20 %, 29 % vs. 15 % and 18 % vs. 5 % for cut off points ≥5/9, ≥6/9 and ≥7/9, respectively [8]. The same pattern was seen for the current 12 year old boys, with a prevalence of GJH also being about twice as high than the normal population of age-matched boys being 19 % vs. 9 %, 10 % vs. 6 % and 5 % vs. 1 % for the three different cut off points, respectively [8]. However, the current high prevalence of GJH is in line with a previous study of adult, competitive swimmers, with prevalence of 20 % for GJH 4≥/9 [7]. Contrary, a previous study of children participating in swimming, found girls aged 9 to have lower BT score than controls (median 2 vs. 3), with no group differences for girls aged 12, but with boys (aged 9 and 12) displaying higher BT score than controls (median 4 vs. 2, and median 2 vs. 1, respectively) [19]. The high prevalence of GJH in the current study of competitive swimmers may be due to the fact that large shoulder mobility with respect to stroke length is recognised as an advantage for competitive swimmer, thereby being seen more prevalent in this sport for those with GJH, as also previously reported [7].

The current study is in line with previous studies showing the prevalence of GJH increasing by age for girls and decreasing by age for boys [8, 23, 24]. Thus, the choice of cut off points for classification of GJH may vary from ≥4/9 to ≥6/9 within this age group [8, 11, 12, 25]. With cut off point ≥6/9 for GJH, almost 33 % of the current girls are considered to have GJH. This is recognised as a relatively high prevalence compared with a normal child population (mean age 13.8 years), where only 7 % of the girls were classified GJH at this cut off point [25]. It seems difficult to determine a single cut off point for children and adolescents, since joint mobility represents a variable fluctuating condition during maturation [24]. It is therefore recommended that future studies present data of GJH prevalence with different cut off points, in addition to age and gender, as presented in the current study.

The limitations of the current study are mainly the unknown reliability and validity for the AHSA test. Previous studies found excellent reliability for inclinometer measurements of shoulder abduction ROM, when tested in a seated position [26]. The purpose of the current test was to measure shoulder mobility, expressed as AHSA, but as AHSA is a coupled motion performed actively, the outcome may rather indicate the swimmers ability to move the arm against gravity, than the actual and specific shoulder ROM. The concurrent validity of the current clinical AHSA test, therefore, remains unknown. However, AHSA was selected due to its similarity with the swimmers freestyle stroke movements, and as such AHSA represents the most swim-related shoulder mobility test.

Another limitation is, that the GJH group accounted for 2/3 girls, which may hamper generalizability. However, since the prevalence of GJH among girls and women is higher in general, the current group may be well representative of the GJH group [27]. The normative values of the current study are distributed by age, but with the small sample sizes in the 10-year-old group (three subjects), the validity of this age group is hampered. A larger sample of this age group is therefore required in future studies for comparison to establish validity.

The strengths of this study are the standardized protocol of the BT and AHSA test, the supervised training phase of the testers and the large sample size. This study was carried out in young, competitive swimmers, making the results largely relevant for this group.

## Conclusions

A positive association between AHSA and GJH was found in young, competitive swimmers, regardless of sex, age and cut off point. Normative values for AHSA ranged from 40° to 52°, depending on age. Due to lack of shoulder mobility tests in the BT scoring system, the AHSA test seems to be a promising supplemental test, displaying shoulder mobility conditions more precisely.

Future longitudinal studies should test GJH including AHSA as predicting factors for shoulder injuries.

## Abbreviations

AHSA, active horizontal shoulder abduction; BT, beighton tests; GJH, generalised joint hypermobility; PPYCS, physical performance in young competitive swimmers; ROM, range of motion
